# Measuring health of highway network configuration against dynamic Origin-Destination demand network using weighted complex network analysis

**DOI:** 10.1371/journal.pone.0206538

**Published:** 2018-11-01

**Authors:** Sehyun Tak, Sunghoon Kim, Young-Ji Byon, Donghoun Lee, Hwasoo Yeo

**Affiliations:** 1 Department of The Fourth Industrial Revolution and Transport, The Korea Transportation Institute (KOTI), Sejong, Republic of Korea; 2 Department of Civil and Environmental Engineering, Korea Advanced Institute of Science and Technology, Daejeon, Republic of Korea; 3 Department of Civil Infrastructure and Environmental Engineering, Khalifa University of Science and Technology, Abu Dhabi, UAE; Universitat Rovira i Virgili, SPAIN

## Abstract

Ideal configuration or layout of highways should resemble the actual demands for the roads represented by Origin-Destination (OD) information. It would be beneficial if existing highways can be evaluated for their configurational fitness against the current demands, and newly planned highways can carefully be designed in terms of their layouts and topologies that would reflect the demands. Analysis techniques used for complex networks in the matured field of network theory can be applied for the highway layout health monitoring against the current OD information. This paper proposes a methodology of measuring the fitness of existing highways by comparing their structural configuration against conceptual OD networks using well-established techniques in network theory for complex networks. In the first phase, this paper conducts an empirical analysis and finds that both structural highway network and OD network follow the “power law” distribution as they are weighted by capacity and traffic volume respectively. It is also found that the power law coefficient of the OD network dynamically changes throughout the day and week. In the second phase, a noble methodology of weighting and measuring the health, of structural highway networks against OD networks by means of comparing their power law coefficients is proposed. It is found that the proposed method is effective at detecting deviations from ideal structural configurations associated with actual demands.

## Introduction

Transportation networks, especially highway networks are considered as permanent investments that are extremely expensive in building, maintaining and modifying them. Therefore, a careful and continuous monitoring of such crucial infrastructures is mandatory for the society. Many studies on transportation network focus on analysing the topological properties of a transportation network such as ringness, webness, circuitness, degree centrality, closeness centrality, betweenness centrality, straightness centrality, and clustering [1–6]. They analyse specific connection patterns of the road network to abstract the properties of a complicated transportation network structure because it is helpful for not only to understand the temporal and spatial changes of the transportation network but also to provide a guideline for road constructions. To estimate the properties of large-scale transportation networks in an efficient way, many empirical studies have attempted to conduct a case study based on the railway, urban, aviation, and maritime transportation networks [7–11].

Recently, the researches related to transportation networks have shifted their focus from topologic and geometric properties to large-scale statistical properties from a point of view of complex network theory [12]. In a complex network with the “power law” distributions, which is known as a scale-free network, the degree of a node indicates the importance and the node with a high degree is considered as critical [13,14]. By identifying critical components (nodes and links) in a network, the potential failure of a component can be prevented. Furthermore, if the distributions of a transportation network are identified beforehand, the evolutional direction of transportation network can be predicted and the heterogeneity of transportation network can be estimated [2,15,16]. For these reasons, different kinds of transportation networks such as air network [17–19], railway network [20,21], public transportation network [22,23], and highway network [24] have been studied. In most of these studies, transportation networks show small-world properties with different degree distributions for each network. For instance, for the airport networks in India and China, the degree distributions are found to follow the power law[17,18]. The degree distribution of Polish public transportation network and that of Indian railway network follow the exponential law [20,22]. The South Korean highway network has a heavy tail with relatively more homogeneous characteristics compared to other transportation networks such as public transportation network and airport transportation network [24]. The previous studies, which focus on the transportation networks based on statistical properties from a point of view of complex network theory and graph theory, analyse the structure of transportation networks without any considerations on the traffic flow and traffic demand on the network, even though the traffic demand and network structure are mutually affected.

Traffic flow and traffic demand on a network shape the network structure in the long run, and instantaneous travel demands reflect the traffic flow. With this background, some previous studies evaluate the characteristics of transportation networks in accordance with the relationship between the desired properties of network structure perceived by road users and the properties of transportation network [1,2,15,16,25–28]. They find the optimal network and measure the efficiency and continuity of transportation network based on the given traffic demand. In these studies, the annual traffic volume is used as the traffic demand on the networks. However, the transportation network is also designed to solve temporal traffic problems such as congestion, which recurrently occurs by the peak demand in a certain period. There is a need for an analysis to identify the relation between the short-term demand change and the properties of transportation network because congestion induced by the short-term peak demand significantly affects the performance of transportation network and the satisfaction of the transportation system users.

Only a few studies have considered statistical properties of Origin-Destination(OD) demand networks based on actual travel patterns [29,30]. In general, traffic demands are not consistent throughout a day or months, and the congestion occurs due to temporal inconsistencies between the designed properties of physical transportation network and desired properties of network structure perceived by road users. Therefore, it is important to study the effect of the temporal inconsistencies on performances of the transportation network. This paper proposes a noble, comprehensive and system-wide approach in evaluating the fitness or health of existing highway network configurations. In the first phase, we explore the relationship between the underlying topological structure of highway systems and the dynamics of demand from a point of view of complex network theory. In the second phase, we analyse the effect of this relationship on the network performance. This study aims to seek for possible solutions for controlling an entire highway network at the state-level and to give guidance for constructions of newly planned highways. We expect that the results of this study provide the foundation to improve the usage of a highway network.

## Analysis approach

In order to assess the fitness of current physical transportation network against the conceptual road network perceived and used by the actual demand of people, structural highway network and hypothetical OD network are compared using the complex network theory. A road network can be modelled with different approaches by taking into account factors including existing physical structures, number of lanes, actual usage, and traffic volume. In this paper, four different types of road networks are conceptualized of which two of them are inspired by the physical structure of the roads and the other two are hypothetical road networks based on actual trips taken by the demand in forms of O-D pairs.

The structural highway network can be classified into two: conceptual road network (CN) and physical road network (PN). CN is the network that represents only the structural connectivity relationship among road links and nodes without the consideration of the number of lanes on each road link. On the other hand, PN considers the number of lanes on each road link in addition to the mere connectivity status of the physical network, which results in changes of degrees of the nodes in the network. CN can be seen as a non-weighted structural road network without consideration of link properties and PN is seen as a weighted structural road network with link properties considered. Second, the hypothetical OD network is also classified into two: movement network (MN) and volume-weighted network (VN). MN is the network that represents the actual travelled roads by the people from origins and destinations. In MN, each OD pair is generated if at least one person has travelled from the origin to the destination regardless of the traffic volume. On the other hand, VN considers the actual traffic volume on the generated OD pair. MN is seen as a non-weighted OD network and VN is seen as a weighted OD network. In order to reflect realistic physical road network and actual demands for assessing the current fitness of current infrastructure for the actual O-D trips, PN and VN are used to identify the contributing factors that affect the performance of a highway network.

Throughout such procedure, we attempt to show three aspects of highway networks. First, this study will newly show the power law relation between the frequency of a degree and the degree of a node in the road network. The degree of node i is calculated as $d_{i}=\sum_{j}^{N}a_{ij}w_{ij}$, where if nodes i and j are connected, a_ij_ = 1, and if not, a_ij_ = 0. N is the total number of nodes in a network and w_ij_ is the weighting factor between nodes i and j.

Existing studies [31,32] on transportation networks show that the degree distribution of a road network appears as a bell shape. However, the weighted degree distribution of a road network actually follows the power law when the number of lanes in each road link is considered. Second, this study will analyse a hypothetical OD network based on real-world data and show the power law relation between the frequency of a degree and the degree of a node in the OD network. Unlike the existing studies on road networks, the studies on OD network are rare due to the difficulties in obtaining data for a large area. Therefore, the studies on the effect of dynamics of an OD network on the performance of existing structural road networks are also rare. Third, this study will propose an evaluation method for the performance of an entire highway network. Existing researches have practiced mostly based on the assumption that the network performance is strongly related to the local traffic demand or local configuration of the network, and the performance level was generally represented by the summation of the median travel time or total travel time of the network. Therefore, most of the studies analysed the network based on the road link performance or effect of the link closure on the network performance. In this study, we present the characteristics of the OD network of a whole road network at the state-level by using a singular value, and then we evaluate the road network performance based on the measurements that represent the structural road network and hypothetical OD network.

In order to derive the CN, PN, MN, and VN for the analysis, we use the databases of Korea highway system collected in 2015, which are public sources provided by Korea Expressway Corporation. To derive the degree distributions of the CN and PN, we use the network structural database in 2015 that contains information on the number of lanes on each highway link, length of each link, and the locations of tollgates (TGs) and interchanges (ICs). The number of nodes in the Korea highway network is 614, and the degree of each node varies from 1 to 4. Both the non-weighted (CN) and weighted (PN) degree distributions of the structural highway network in Korea are derived using the degree values of each node and number of lanes on each road link. To derive the degree distributions of the MN and VN, we use the database of Toll Collection System (TCS), which contains information on the number of vehicles travelled for each OD pair collected hourly in 2013. Using this database, both the non-weighted degree distribution (MN) and the weighted degree distribution (VN) in Korea highway system are derived at every 4-hour interval.

## Analysis of physical highway network

**Fig 1:**
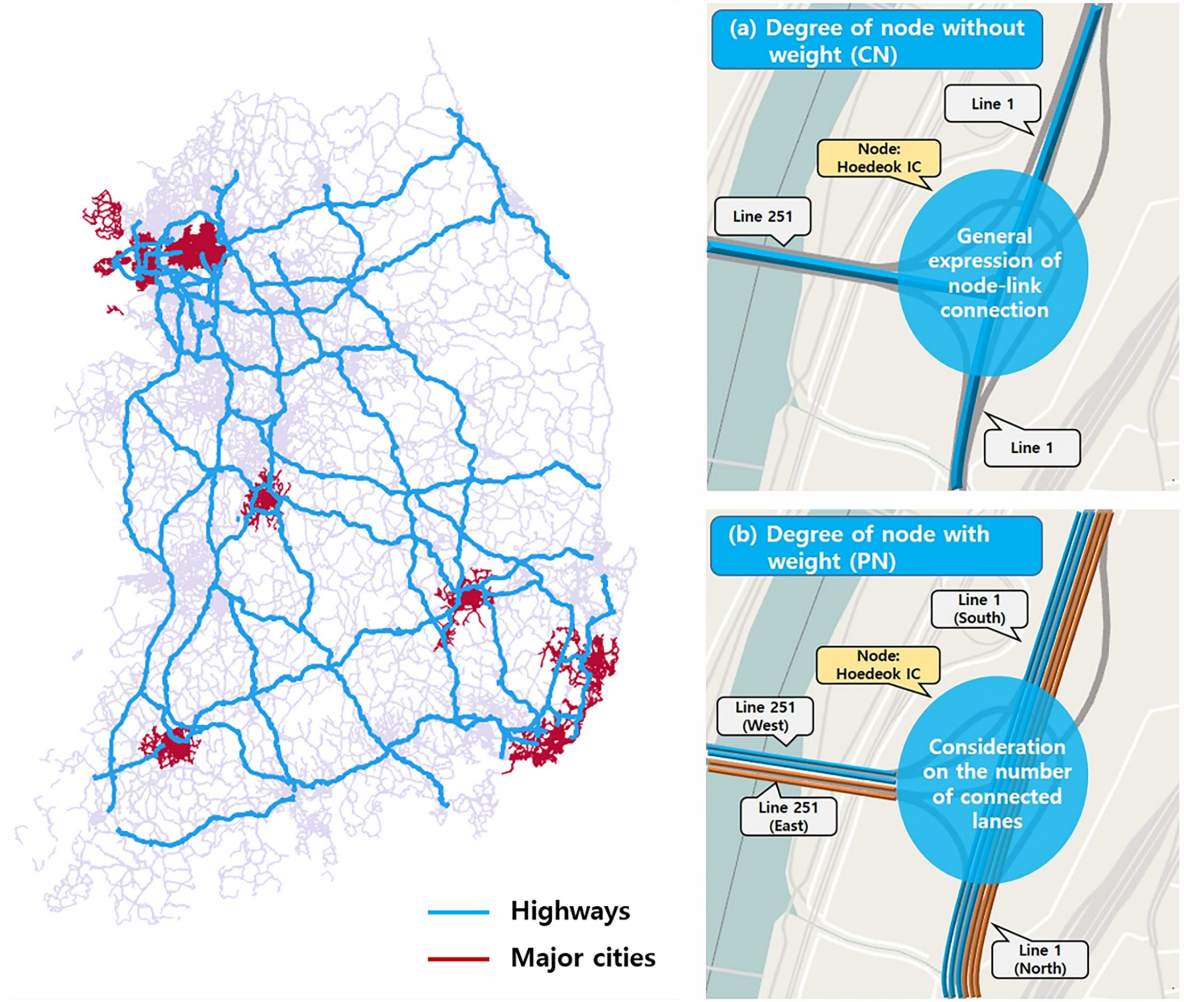
Road network of Korea highway. (Drawn by authors using QGIS program, data source: Ministry of Land, Infrastructure and Transport (MOLIT), the data are free source for everyone, the authors did not have any special privileges that others would have in obtaining the data).

Fig 1 shows the road network of Korean highway system, which is the structural network used in this study. As shown in the figure, the tollgates and interchanges on the road network are considered as the nodes. Tollgates (TGs) are the starting and ending points of each highway line. They are also the entering points to the highway or exiting points from the highway. Interchanges (ICs) are the intersecting points of two or more lines of the highway. In the road network of Korea highway, the lines connecting TGs and ICs are considered as the links. Based on given conditions, we propose to analyse the structural road network and draw the degree distribution in a log-log plane.

At first, we draw un-weighted degree distribution of the road network of Korea highway. At this point, note that the general (un-weighted) degree distribution of a road network has some weakness in representing the nature of the road network. In the social network such as Facebook and Twitter, the link connection can easily be generated by just clicking a button on a computer and this action relatively does not cost much for connecting the link between two nodes. However, in a physical road network, constructing a new link between two nodes is associated with high construction cost. Particularly in a highway network system, the capacity of a road link can be increased by expanding the width instead of building a new link. The number of lanes in a link is increased by the width expansion. In order to reflect this nature in the degree distribution of road networks, we give weighting factors to a road link based on the number of lanes on the link. The number of lanes is considered to represent the road link capacity. In fact, if we were to consider the traffic flow theory more deeply, the capacity of each road lane may be a bit different. However, such deep consideration is not reflected because it does not match the scope of this study. Thus, homogeneous values are assumed for the capacity of all lanes in a link, and the number of lanes in a link represents the degree of the road capacity. As an example, Hoedeok IC has the degree of three in un-weighted degree distribution as shown on the upper right side of Fig 1. This is the example of the conceptual network (CN). In weighted degree distribution (bottom right corner of Fig 1), the degree of the node is 10, which is the summation of the number of lanes in all links that are connected to the node. This is the example of the physical network (PN).

The un-weighted degree (CN) and the weighted degree (PN) distributions of the road network are to be drawn in a log-log plane. In this plot, two aspects are to be analysed. First, we show whether the degree distribution of the road network follows the normal distribution or the power law distribution. Second, if road network follows the power law distribution, we try to find that $f_{d}\propto d^{C_{PN}}$, where f_d_ is the frequency of a degree and d is proportional to the degree of a node to the power of a constant, C^PN^, in the road network.

**Fig 2:**
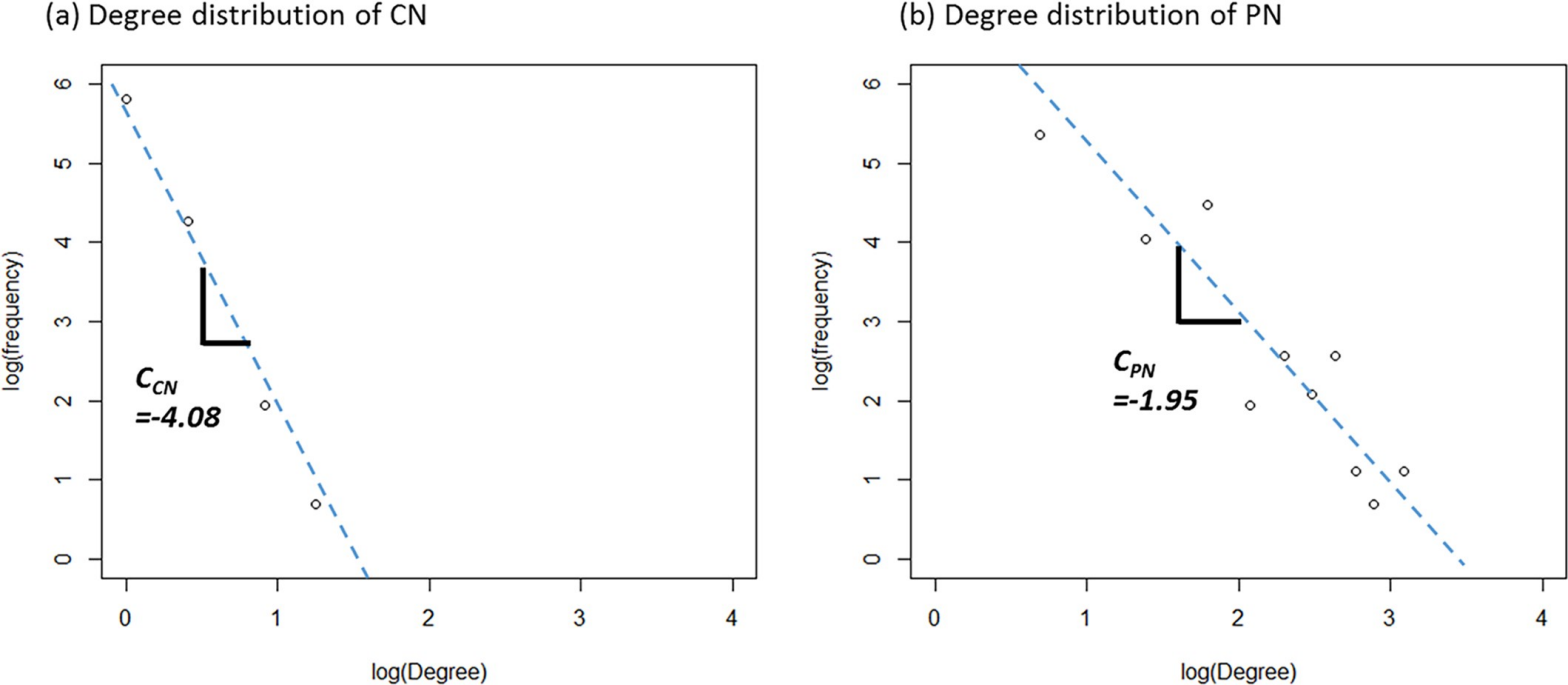
Degree distribution of road network in Korea highway. (Drawn by authors using R program, data source: Korea Expressway Corporation, the data are free source for everyone, the authors did not have any special privileges that others would have in obtaining the data).

Fig 2 shows the degree distributions of the structural Korea highway network. As shown in the figure, the general degree distribution of the structural road network (CN) and weighted degree distribution of structural road network (PN) show the linear relation in the log-log planes with -4.08 and -1.95 coefficient values, respectively. As mentioned above, the degree distribution of CN is not spread over a large range and shows the narrow range of values. It is difficult to say that the coefficient of the general degree distribution represents the nature of the road network reasonably well. The weighted degree distribution of PN shows a wider range of the values compared to CN, meaning that it deals with the various aspects of the network. So, for the network performance analysis, we use the coefficient of the degree distribution of the PN as the representative value for the structural road network.

## Analysis of Origin-Destination demand network

**Fig 3:**
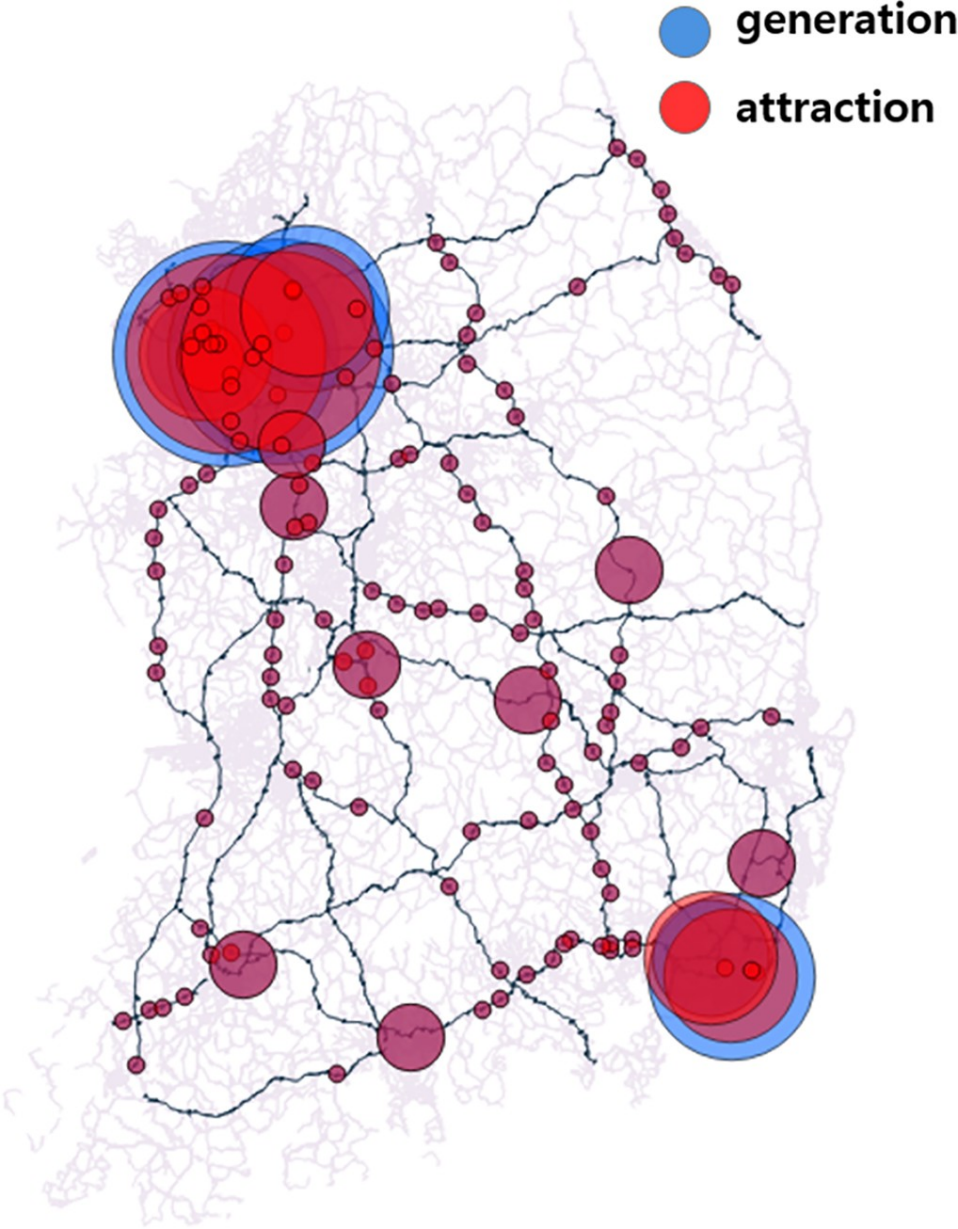
Average daily traffic pattern of Korea highway. (Drawn by authors using QGIS program, data source: MOLIT and Korea Expressway Corporation, the data are free source for everyone, the authors did not have any special privileges that others would have in obtaining the data).

**Fig 4:**
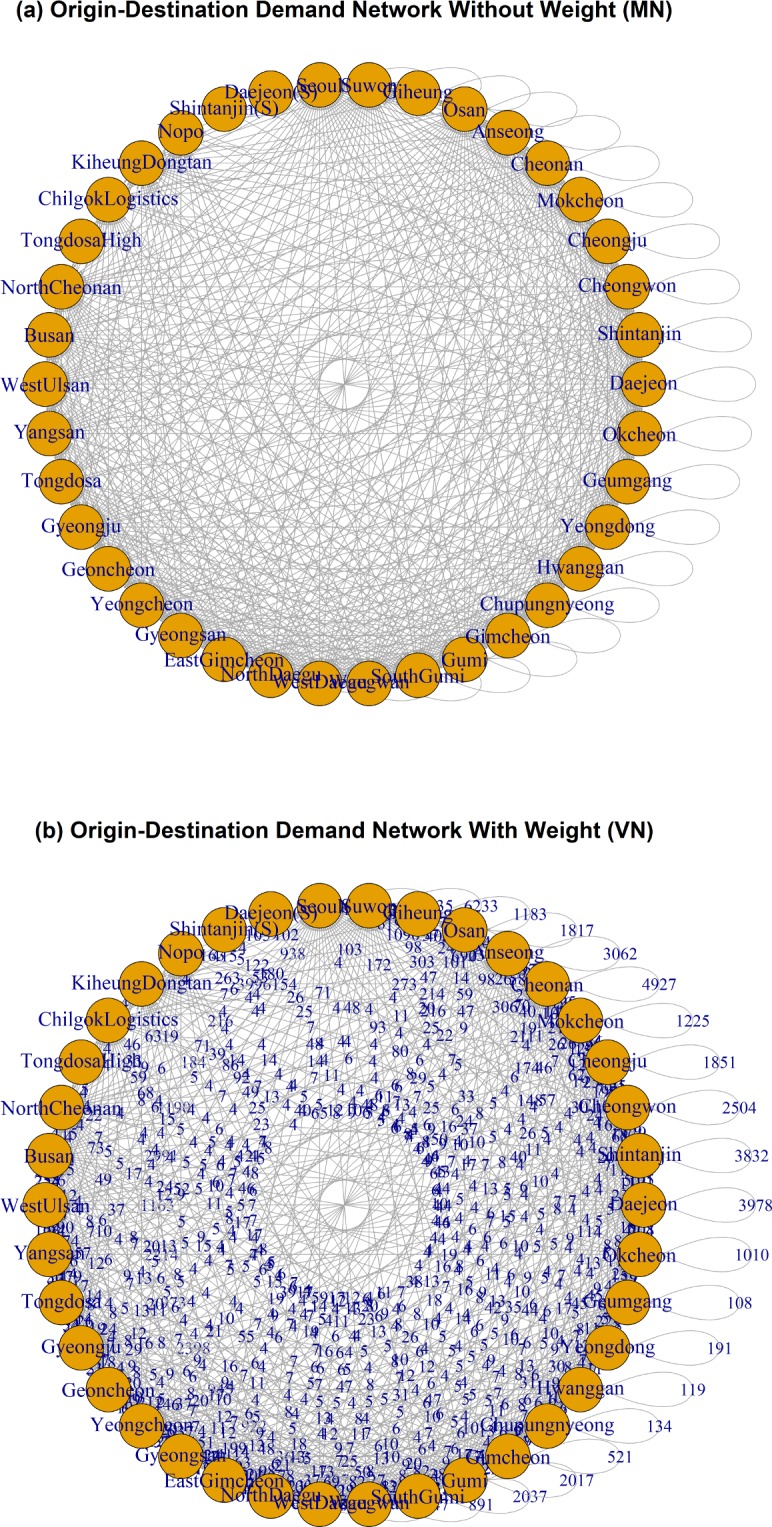
Example of Origin-Destination network. (Drawn by authors using R program, data source: Korea Expressway Corporation, the data are free source for everyone, the authors did not have any special privileges that others would have in obtaining the data).

Fig 3 shows the average daily traffic pattern of Korea highway, which is drawn based on the number of vehicles counted at each TG in 2015. This figure presents the patterns of traffic entering (attraction) and exiting (generation) the local areas at a glance. Fig 4(A) shows an example of the OD network of Korea highway system. In the OD network, each of the TGs is considered as a node, and the connections between TGs are considered as links when there is at least one traveller who moves from node i to node j. When there are no people moving from node i to node j, the link between i and j is not generated in the OD network.

To analyse the OD network, we draw the directed graph as shown in Fig 4(A). Each node is a TG in the highway system and has in-degree and out-degree values. In-degree value is related to how many origins are directed to a destination, and out-degree value is related to how many destinations are directed from an origin. In this method, all links are considered to have the same weight regardless of the amount of people’s movements. This non-weighted OD network is called movement network (MN). Based on the degree distribution of the MN, we analyse the nature of the OD network.

In addition, each link in the OD networks has different demand value for movement. In order to reflect the demand of each link to the OD network, we assign weighting factors to each link based on the OD demand. The Fig 4(B) shows the example of the OD network of Korea highway system that is weighted by the number of travellers moving from node *i* and node *j*. In this study, this weighted OD network is called the volume-weighted network (VN), and the weighted degree distribution of the VN is to be also analysed in a log-log plane.

Unlike the PN, the degree distribution of the VN changes continuously with time. For dealing with this nature of differences, we plan to estimate the coefficient of the distribution of OD network to find that $f_{d}\propto d^{C_{VN}(t)}$, where *f*_*d*_ is the frequency of a degree and *d* is proportional to the degree of a node to the power of constant C_VN_, at time *t*.

**Fig 5:**
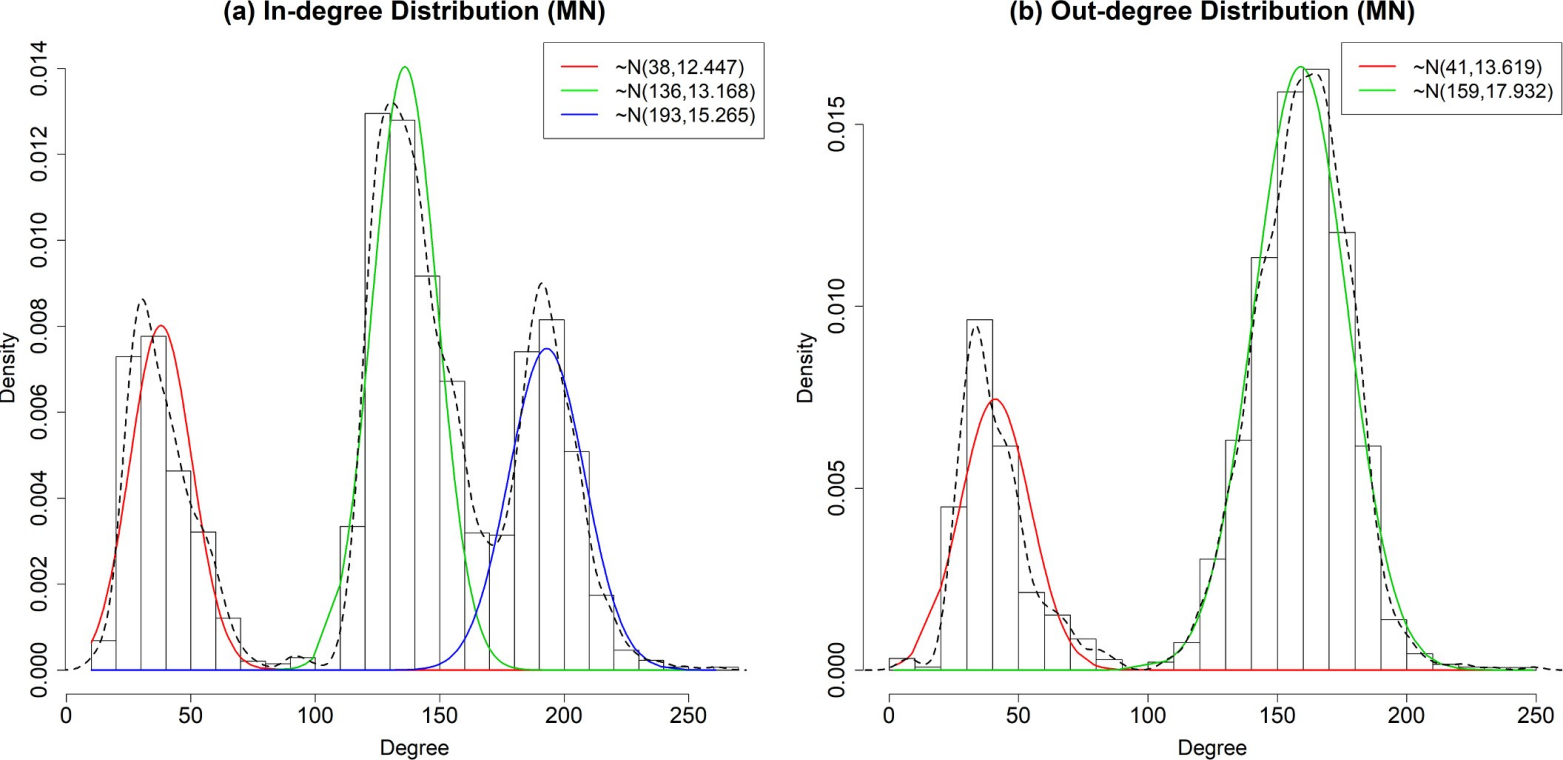
Degree distribution of Origin-Destination network. (Drawn by authors using R program, data source: Korea Expressway Corporation, the data are free source for everyone, the authors did not have any special privileges that others would have in obtaining the data).

Fig 5 shows the degree distributions of OD networks for the in-degree and out-degree MNs, where the density in the y-axis of Fig 5 indicates the average frequency density for each bin in the histogram of degree. As shown in the figure, both the in-degree and out-degree distributions do not show the power law relation. The in-degree distribution shows a mixture of three normal distributions with {(*μ*, *σ*)| (*38*, *12*.*447*), (*136*, *13*.*168*), (*193*, *15*.*265*)}. The mixture model is verified based on the Kolmogorov-Smirnov (K-S) test, the corresponding D statistics and P-value are 0.00078 and 0.937, respectively. For the in-degree distribution, the degree values show certain patterns by the size of the city that is near or within a certain node. As we can see in Fig 5(A), for the nodes near or within small-sized cities, approximately 38 origins are connected to each node. For the nodes near or within medium-sized cities, approximately 136 origins are connected to each node. For the nodes near or within big-sized cities, approximately 193 origins are connected to each node.

On the other hand, the out-degree distribution shows a mixture of two normal distributions with {(μ, σ)| (41, 13.619), (159, 17.932)}. Similar to the in-degree distribution, the mixture model for the out-degree distribution is also verified based on the K-S test. The corresponding D statistics and P-value are 0.00112 and 0.798, respectively. As we can see in Fig 5(B), for the nodes near or within small-sized cities, approximately 41 destinations are connected from each node. For the nodes near or within big-sized cities, approximately 159 destinations are connected from each node.

**Fig 6:**
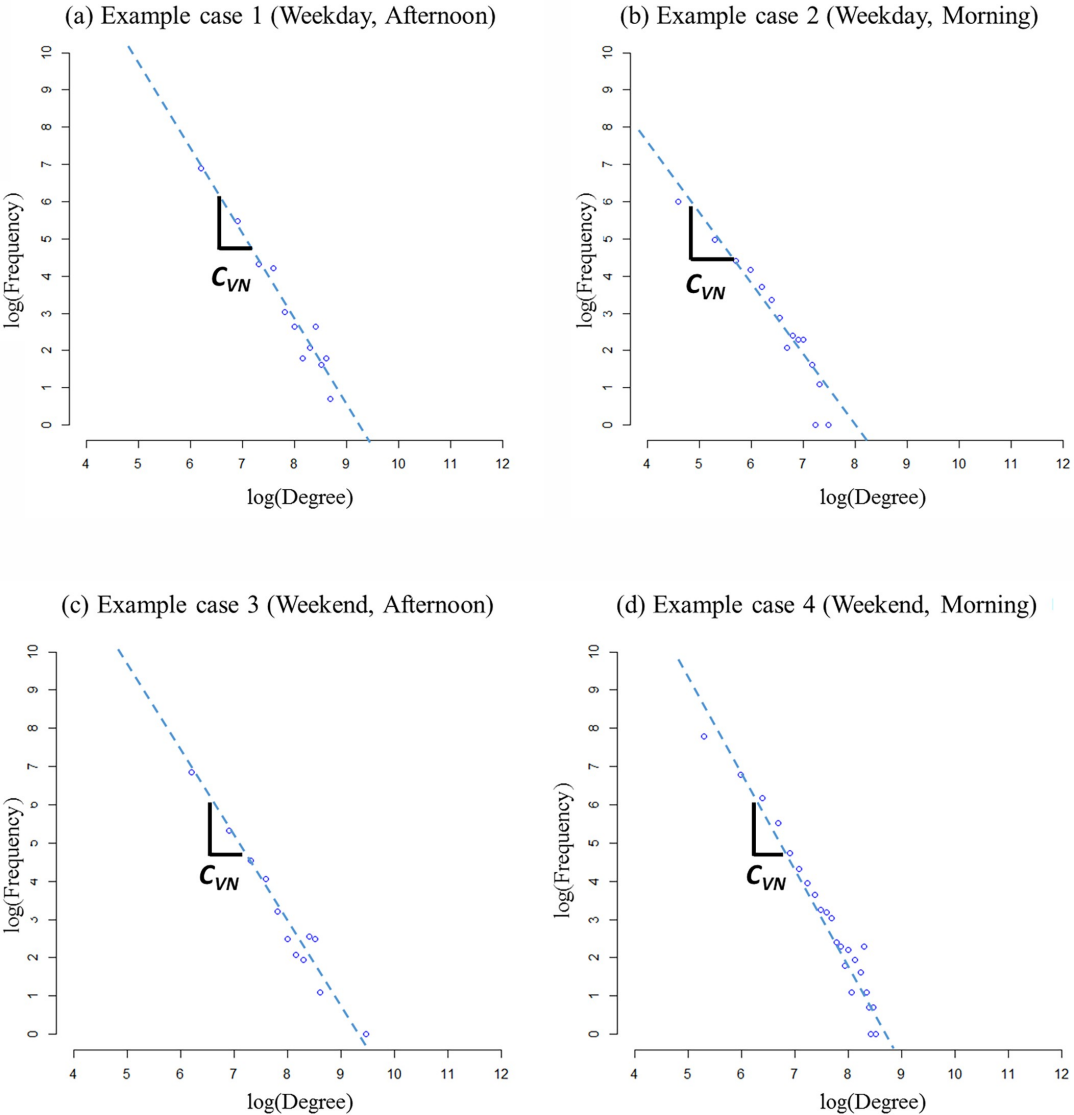
Example cases of the demand distribution of VN during weekday and weekend. (Drawn by authors using R program, data source: Korea Expressway Corporation, the data are free source for everyone, the authors did not have any special privileges that others would have in obtaining the data).

In the case of out-degree distribution, the degree distribution of the nodes near or within medium-sized cities and big-sized cities do not show a clear difference. It means that if a city becomes larger than a certain size, the nodes near the city have a higher tendency to be selected as the destination of trips started from various origins. These results of the MN show that, as a city gets bigger, the number of different OD pairs including the nodes near or within the city tends to increase. The degree distributions of VN show different results as shown in Fig 6. Note that the travel volumes of the OD pairs in the network represent VN. In order to collect the volume of an OD pair, multiple vehicle trips have to begin at the origin and they have to end at the destination. Therefore, VN represents the both in and out degrees of the vehicle trips within the highway system. The general trend of the degree distribution of VN follows the power law distribution. By comparing the distributions during weekdays and weekends, one can observe that the coefficient value of the weekend distribution shows less than that of the weekday distribution because the travel demands are more concentrated in specific nodes during the weekends. This difference is due to the different travel pattern between weekend and weekday. During weekdays, people use the highway system usually for the purpose of commuting and business. So, the travel demands are more likely distributed all over the highway network rather than being concentrated in specific places. On the other hand, during weekends, people usually travel to similar destinations from the various origins. For example, during weekends, many people who live in various places travel to places where they can spend their leisure times. This pattern makes the travel demands be more concentrated in some particular places. So, such a pattern is reflected in the degree distribution of the VN in Fig 6.

The coefficient of VN also varies depending on the time of a day. Fig 6(A) and 6(B) show the distributions of afternoon and morning time during a weekday, respectively. The coefficient in the afternoon is smaller than the coefficient in the morning, meaning that the travel demands in the afternoon tend to be more concentrated in specific places compared to the morning. It is because people show a greater tendency of traveling to specific places, and in this case, the places are the residential areas. In modern cities, the residential areas are usually placed together in specific areas. Hence, most people travel from each of their workplaces to the residential areas in the afternoon, and it causes the travel demand tending to be concentrated in specific areas of the highway network. The time-dependent changes in the demand pattern also occur during the weekend, as shown in Fig 6(C) and 6(D).

For more detailed analyses of trends on the regression coefficients of VNs with respect to day types, including weekday and weekend, several regression analyses are conducted based on the entire OD dataset in 2013.

**Fig 7:**
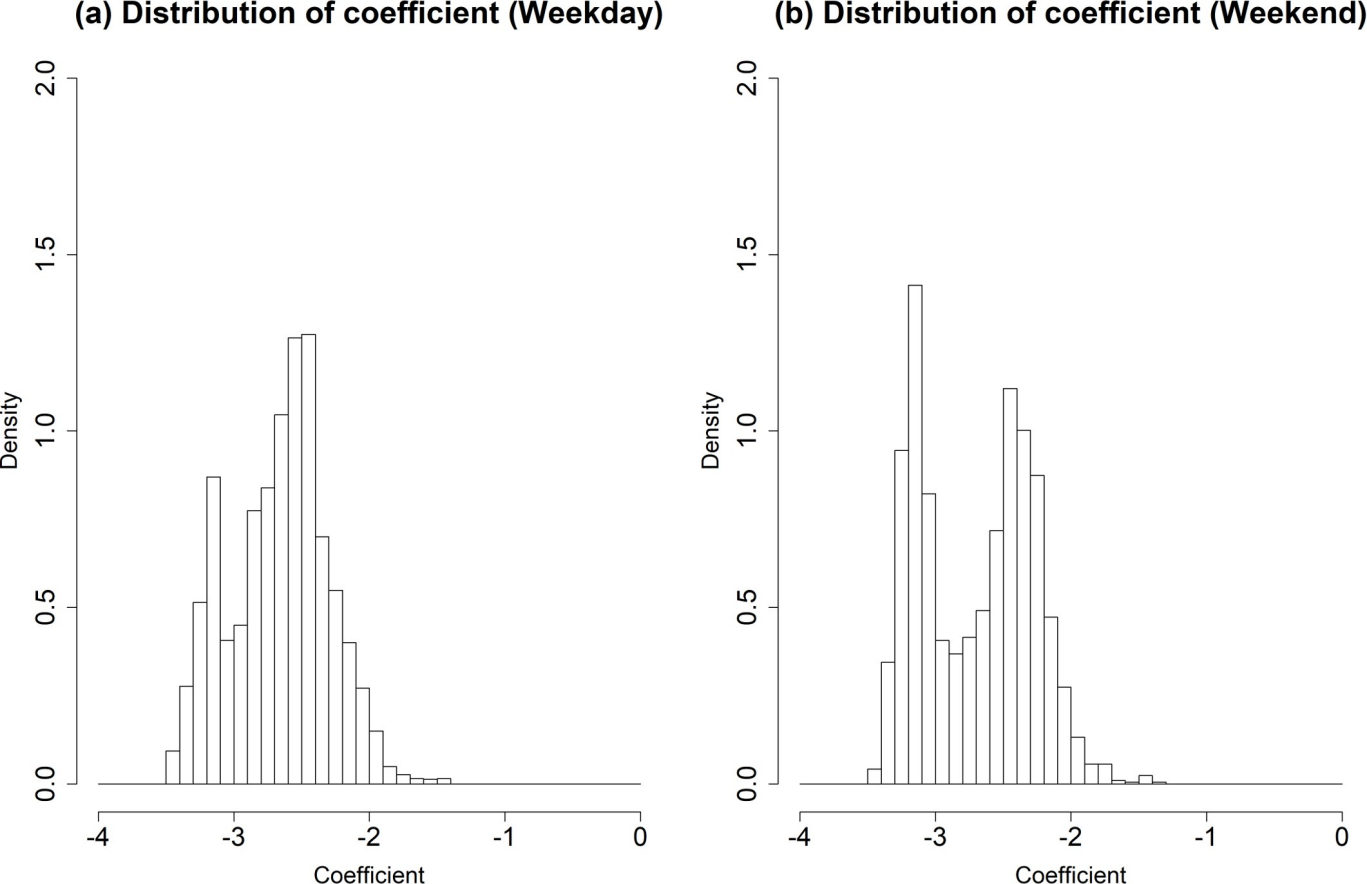
The distributions of regression coefficients of VNs for each weekday and weekend. (Drawn by authors using R program, data source: Korea Expressway Corporation, the data are free source for everyone, the authors did not have any special privileges that others would have in obtaining the data).

Fig 7 depicts the distributions of regression coefficients of VNs for each weekday and weekend, where the density in the y-axis of Fig 7 represents the average frequency density for each bin in the histogram of coefficient. As shown in Fig 7, the maximum density of regression coefficient value in the weekday is observed when the coefficient value is from -3.20 to -3.10, while the maximum density of regression coefficient value in the weekend is found when the coefficient value ranges from -2.40 to -2.30. One can also observe that a greater density value is shown in the coefficient values ranging from -2.40 to -2.30, rather than those ranging from -3.20 to -3.10. These imply that the travel demands are more concentrated in certain OD pairs during the weekends compared to the weekdays’ pattern, which agrees with the research finding of the previous analysis, as shown in Fig 6. In other words, this result shows that the travel demand in the weekday has more evenly distributed OD pairs than in the weekend, while the travel demand in the weekend tends to be concentrated in specific places.

**Fig 8:**
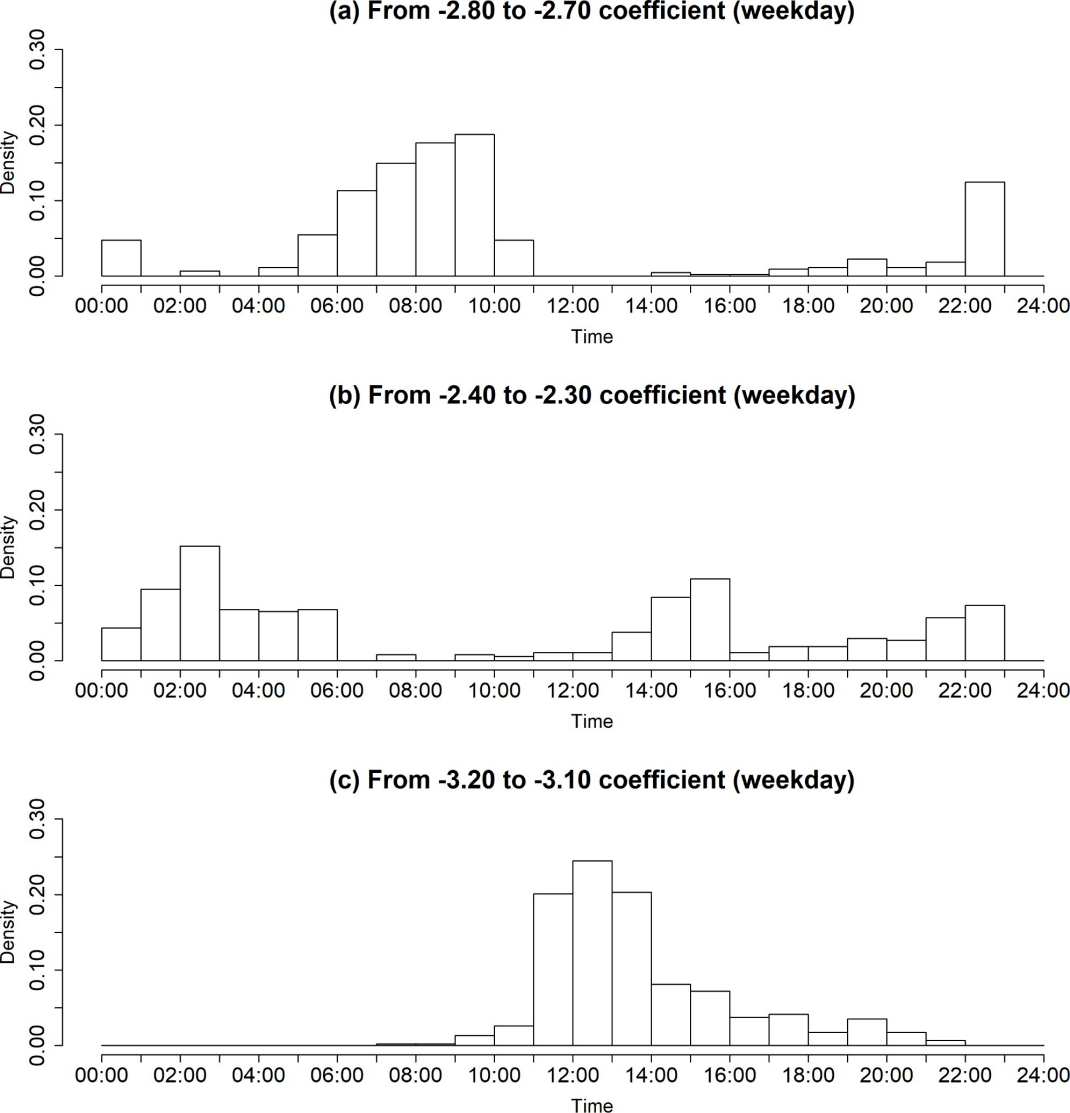
The weekday density trend according to the time evolution with different regression coefficient values. (Drawn by authors using R program, data source: Korea Expressway Corporation, the data are free source for everyone, the authors did not have any special privileges that others would have in obtaining the data).

Based on the observations in Fig 7, Fig 8 shows the weekday density trend according to the time evolution with a specific range of regression coefficient values, where the range of regression coefficient values includes (a) from -2.80 to -2.70, (b) from -2.40 to -2.30, and (c) from -3.20 to -3.10. As seen in Fig 8, it can be found that there are several points in time for a dramatic change of regression coefficient value. As shown in Fig 8(A), one can observe that the VN during the morning peak hour from 5 a.m. to 9 a.m. has the coefficient values ranging from -2.80 to -2.70. The coefficient value decreases to approximately -3.10 as the commuting demand decreases overall. After a while, the VN has a specific value of regression coefficient ranging from -2.40 to -2.30, as shown in Fig 8(B). We conjecture that the reason behind the concentration is due to the increased travel demands for logistics services. One can also find that the VN with the coefficient values ranging from -2.40 to -2.30 has the maximum density value around 3:00 a.m., while the VN with the coefficient values ranging from -3.20 to -3.10 shows the peak value around 12:00 a.m., as described in Fig 8(C).

**Fig 9:**
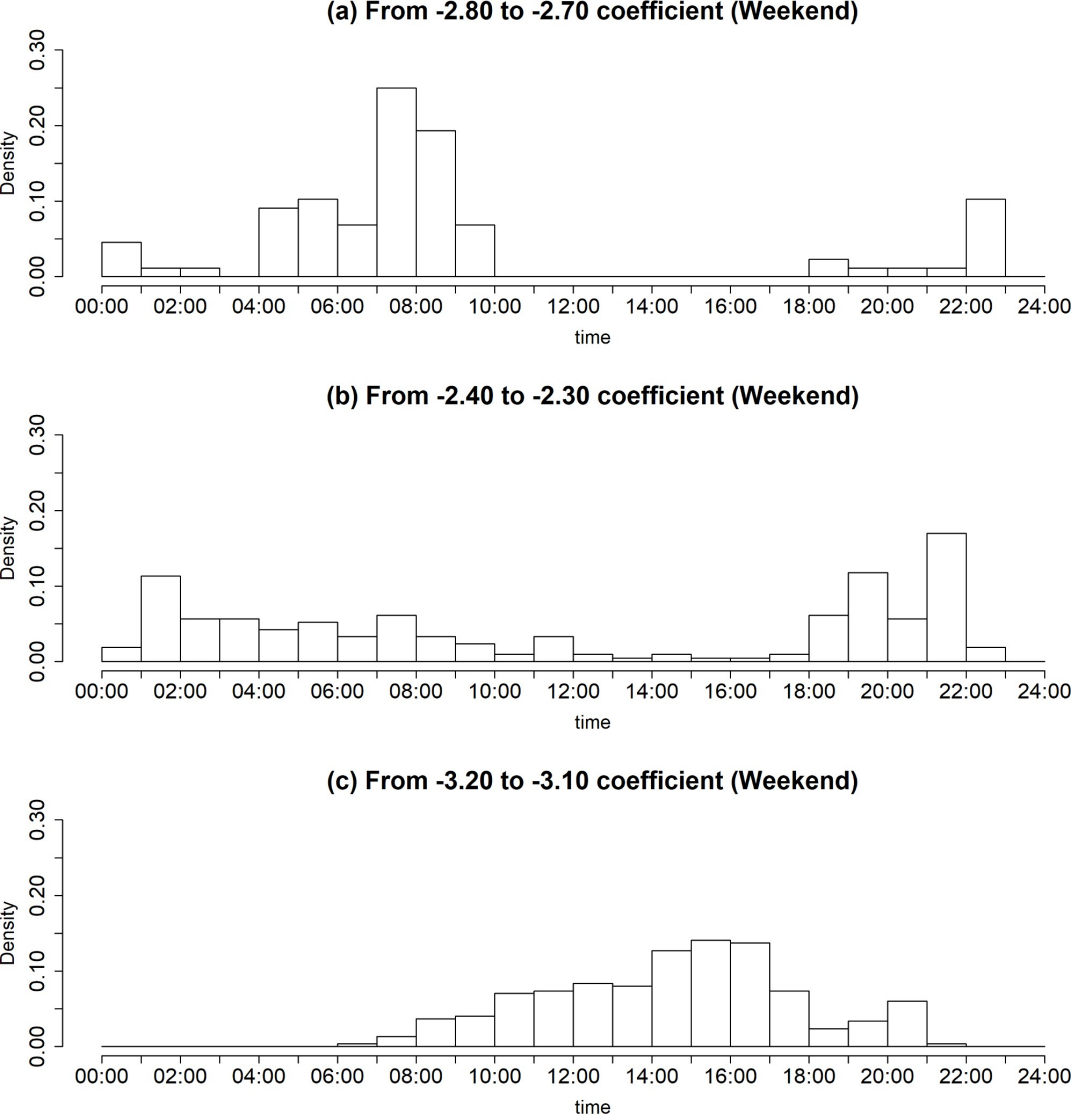
The weekend density trend according to the time evolution with different regression coefficient values. (Drawn by authors using R program, data source: Korea Expressway Corporation, the data are free source for everyone, the authors did not have any special privileges that others would have in obtaining the data).

Such trends can be also observed in the weekend, as depicted in Fig 9. However, distinct from the observation in the weekday’s travel pattern, we find that there is a significant difference in the density between the VN with the coefficient values ranging from -2.40 to -2.30 and the VN with the coefficient values ranging from -3.20 to -3.10. In other words, unlike the weekday’s travel pattern that people use the highway system usually for the purpose of commuting and business, the OD travel demand is changed with the tendency toward dichotomization.

**Fig 10:**
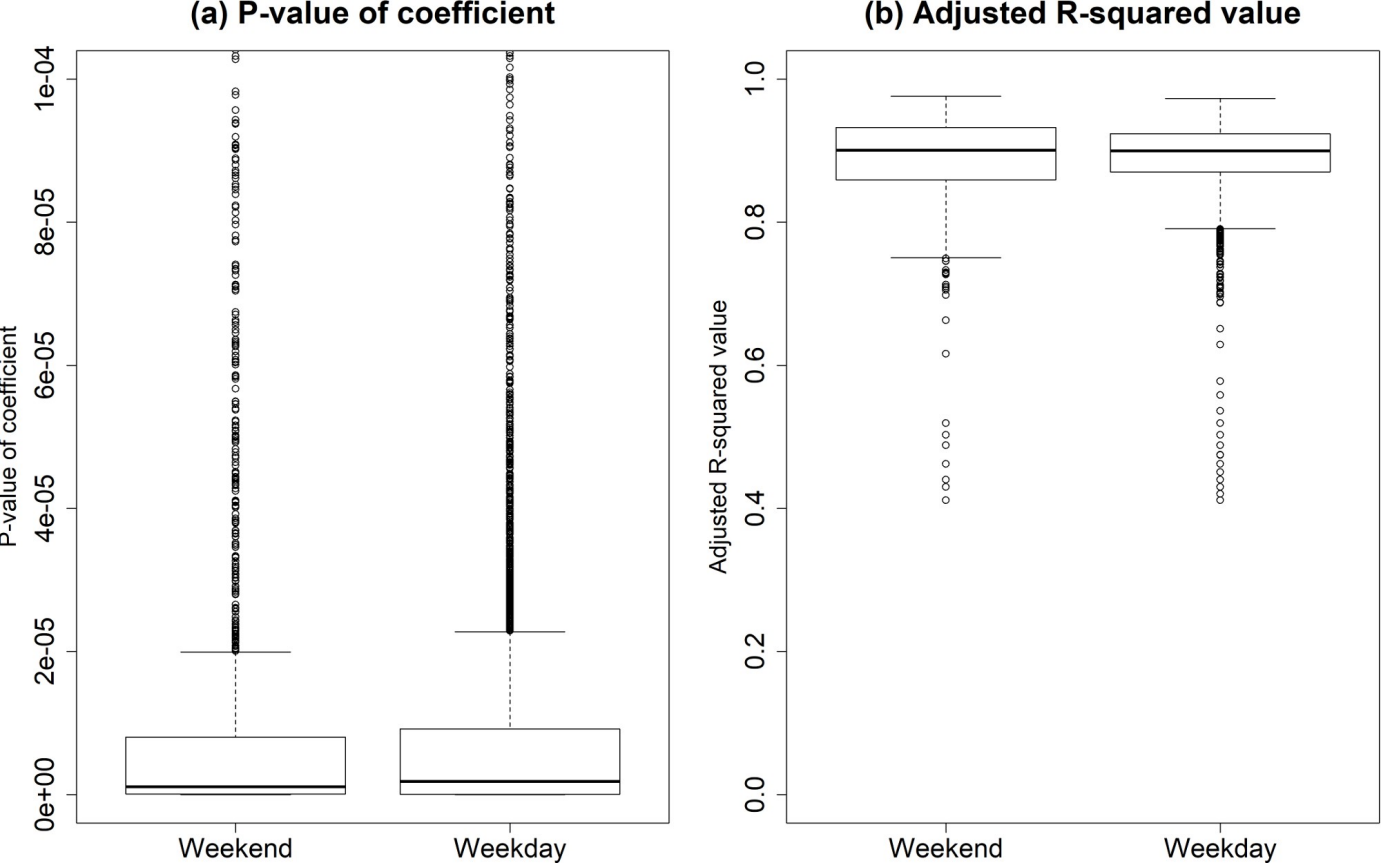
The goodness-of-fit of each regression coefficient of VNs with respect to day type. (Drawn by authors using R program, data source: Korea Expressway Corporation, the data are free source for everyone, the authors did not have any special privileges that others would have in obtaining the data).

As seen in Fig 10, we observe that the maximum P-values of the weekend and weekday are 1.992*10^−5^ and 2.275*10^−5^, respectively. It is also seen that the corresponding minimum values of adjusted R^2^ of the weekend and weekday are 0.75 and 0.791, respectively. Not only the maximum P-values show much less than the significance level, but also the adjusted R^2^ values are high enough. This indicates that there is not sufficient evidence to reject the alternative hypothesis that the corresponding coefficient value is not equal to 0. Therefore, we can conclude that the results of the analyses are statistically significant.

## Network performance analysis based on the weighted networks

The performance of a highway network can be measured with various values such as travel time, frequency of congestion, and delay. In this study, we use the summation of the median travel time from origin *i* to destination *j* for measuring the performance of a highway network at time *t* as follows:

$$Network\;Performance\;(t)=\sum_{ALL\;i,j}{TT}_{median,i,j}(t)$$ (1)

Median travel time indirectly represents the congestion level of a highway network and shows the efficiency of the network for each given OD pair under a given total demand on the network. When a congestion occurs, which is caused when OD demand is larger than the road capacity, the median travel time becomes larger compared to the median travel time in free flow state. When OD demand is lower than the road capacity, the median travel time is the same with the median travel time in a free flow state. Therefore, by summing the median travel times of all links, the performance of the entire network can be estimated. We use the median travel time values provided in the database of Korea highway system for the performance evaluation, which are computed by the Korea Expressway Corporation. The median travel time is computed based on the travel time values of multiple vehicles that have finished the trip between origin *i* and destination *j*. The travel time of each vehicle is computed by calculating the difference between the inbound timestamp at origin *i* and outbound timestamp at destination *j*. The timestamps are recorded at all tollgates by the highway TCS.

With this performance measure, we plan to explore the causal factor degrading the highway performance among overall demands on the highway network and concentration of demands in a specific part of the network. At first, to verify the relationship between the total demand and network performance, we plan to analyse whether the following relation is satisfied or not.

$$\sum_{All,i,j}{TT}_{median,i,j}(t)\propto \sum_{All,i,j}D_{i,j}(t)$$ (2)

where *D*_*i*,*j*_ is the collected number of people travelled from node *i* to *j* at time *t*. In many studies, the assumption that the congestion is proportional to the total demand on a road network is general and many control methods try to mitigate the congestion by reducing and distributing the total demand on a network. In this study, we plan to verify this relation also.

**Fig 11:**
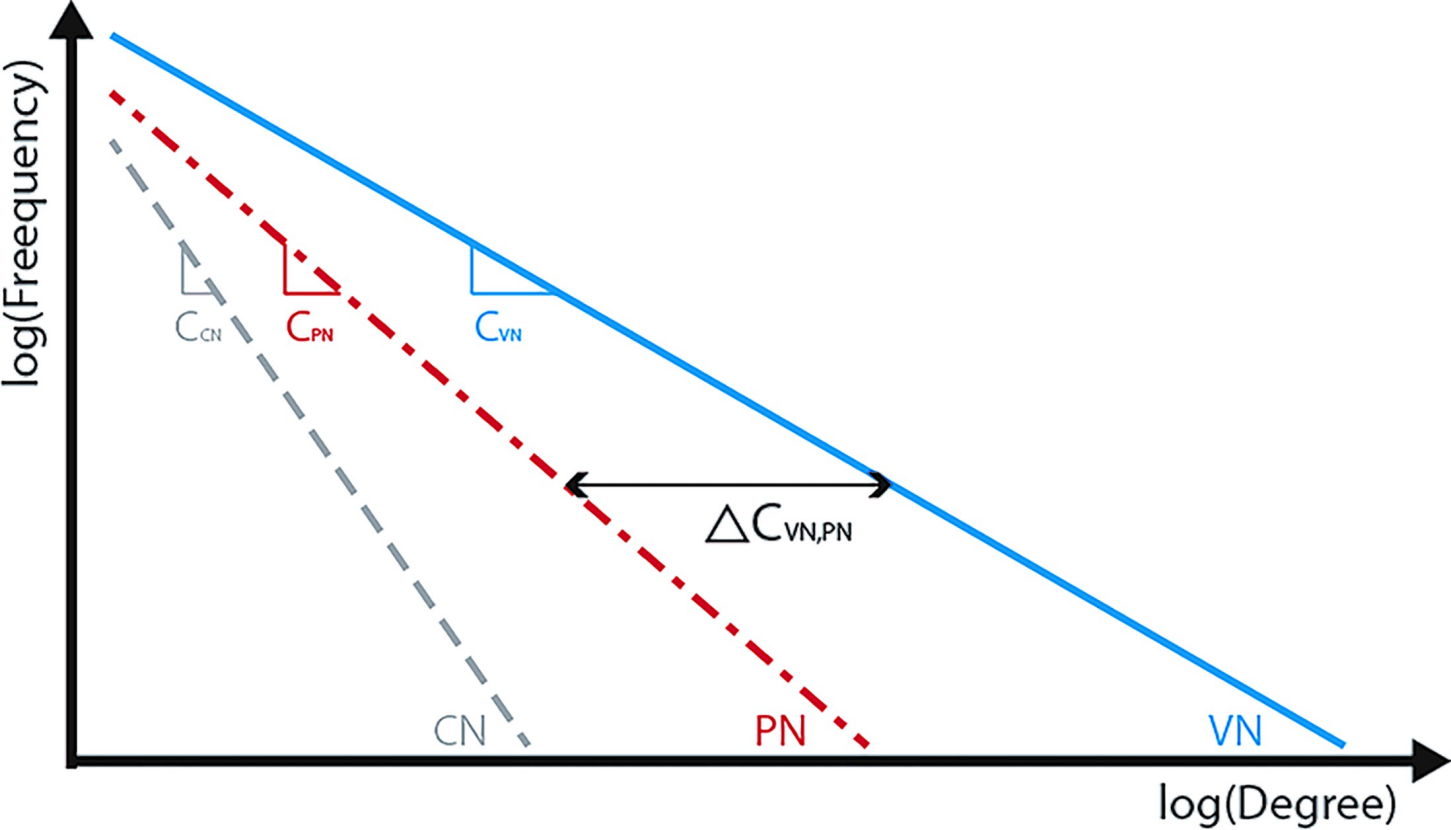
Estimation method of coefficient difference between PN and VN. (Drawn by authors using R program).

Fig 11 shows the conceptual diagram of three different networks. In the figure, C_CN_, C_PN_, and C_VN_, represent the power law coefficient values of the conceptual network (CN), physical network (PN), and volume-weighted Network (VN) respectively.

As shown in the figure, the difference between PN and VN can be estimated by the power law coefficient. This difference represents the imbalance of road configuration and road users’ desires to travel on the highway network. Considering the continuous changes in the demand, the coefficient difference is expressed as follows.

$$\Delta C_{VN,PN}(t)=C_{VN}(t)-C_{PN}$$ (3)

The difference between the degree distributions of a PN and VN can show the road performance. To figure out the effect of this difference on the road performance, we plan to explore the relationship between the power law coefficients of a network and the performance of a network based on whether the following relation is satisfied or not.

$$\sum_{All,i,j}{TT}_{median,i,j}(t)\propto \Delta C_{VN,PN}(t)$$ (4)

The power law coefficient of the network represents the dispersion of the degree. The low absolute value of the power law coefficient means that the demand is concentrated in a specific area of a network. Conversely, the high absolute value of power law coefficient means that the demand is almost uniformly distributed in the entire network. In this context, the difference of the power law coefficient between a PN and VN can show the difference of dispersion between the two networks, and such relation is set based on the assumption that congestions are caused by the difference between people’s desired movement patterns and the physical configuration of a road network.

The total demand and coefficient difference show the different aspects of demand for the network. Total demand represents the entire quantity of travel desires on a network and the coefficient difference represents the difference between the characteristics of the traffic distributions in VN and PN. We also explore the following relation to consider these two different aspects of the road network.

$$\sum_{All,i,j}{TT}_{median,i,j}(t)\propto \Delta C_{VN,PN}(t)*\sum_{All,i,j}D_{i,j}(t)$$ (5)

The coefficient of the VN dynamically changes and the difference of the coefficient between the VN and PN changes as well. Based on this phenomenon, we analyse the changes of the coefficient difference and total OD demand respect to the sum of the median travel time.

**Fig 12:**
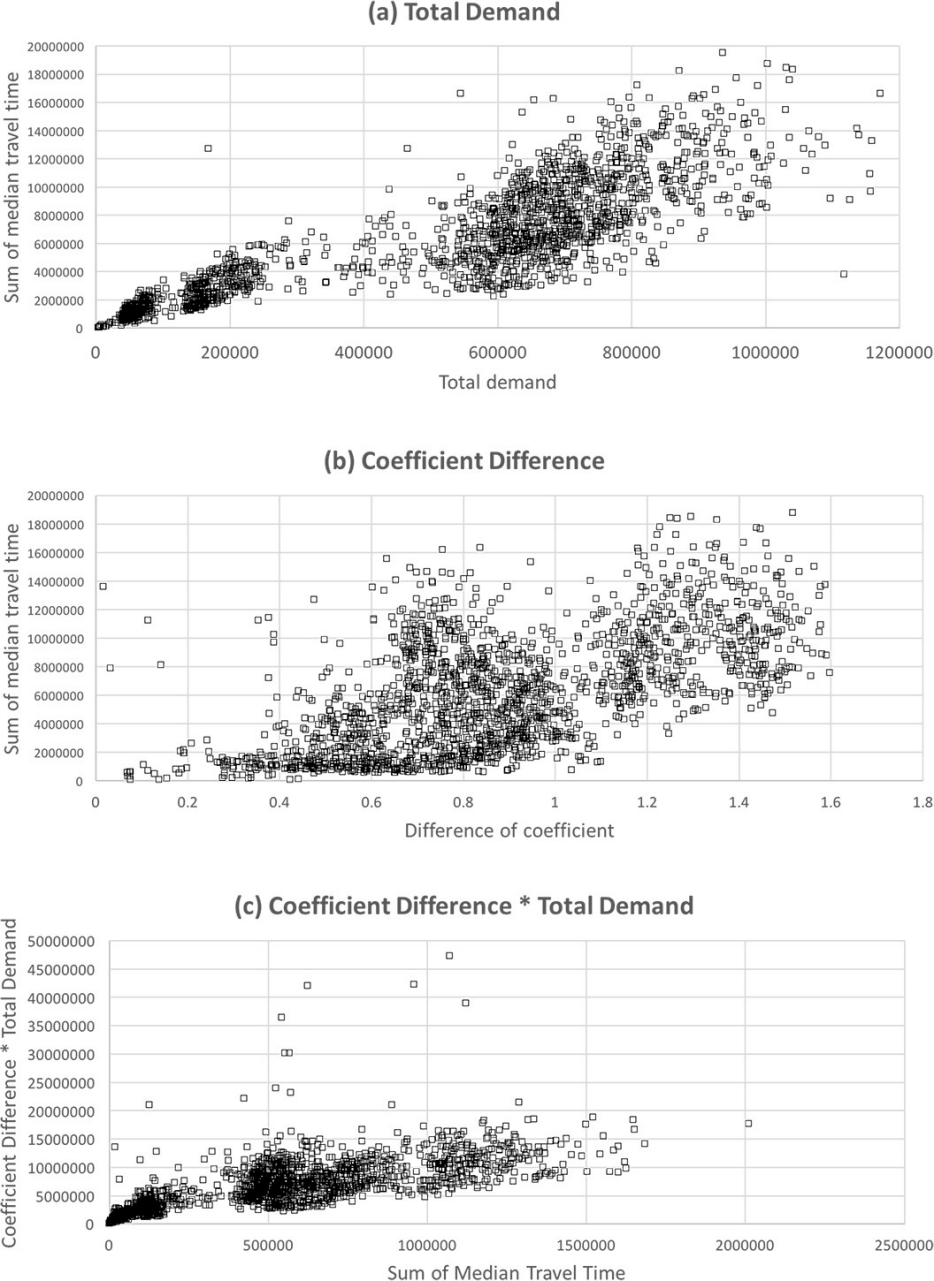
The effect on the road performance. (Drawn by authors using R program, data source: Korea Expressway Corporation, the data are free source for everyone, the authors did not have any special privileges that others would have in obtaining the data).

Fig 12(A) shows the relationship between the total demand and sum of median travel time. As we can see in the figure, when the total demand increases, the sum of median travel time also increases. This shows that the network performance is degraded as total demand increases. It is noticeable that there are two clusters in the relationships and each of them shows different increasing slope from each other. When total demand is small, the relationship is clearer with less sparse data points, whereas the relationship is sparser when the demand is big.

Fig 12(B) shows the relationship between the difference of coefficient and the summation of the median travel time for each OD demand. As shown in the figure, the sum of the median travel time increases as the difference of coefficient increases. This means that the performance of a road network is affected by the coefficient difference as well. It can be noted that the data points showing the relationship are sparser compared to the relationship between the total demand and the sum of median travel time. Using only the coefficient difference may not be appropriate when evaluating the network performance.

Fig 12(C) shows the product of coefficient difference and total demand respect to the sum of median travel time. As we can see in the figure, when we considered the two aspects together, it shows a much clearer relationship that is linear. In addition, we do not see the phenomenon of clustering in the relationship that is shown in Fig 12(A) anymore. Based on this result, it seems that using the product of the two aspects can more efficiently evaluate the performance of a road network. There are some outliers in the relationship, and it is assumed that these outliers occur due to the factors that are related to total demand, such as special cases like accidents and incidents. Still, these outliers have to be investigated in further studies.

**Fig 13:**
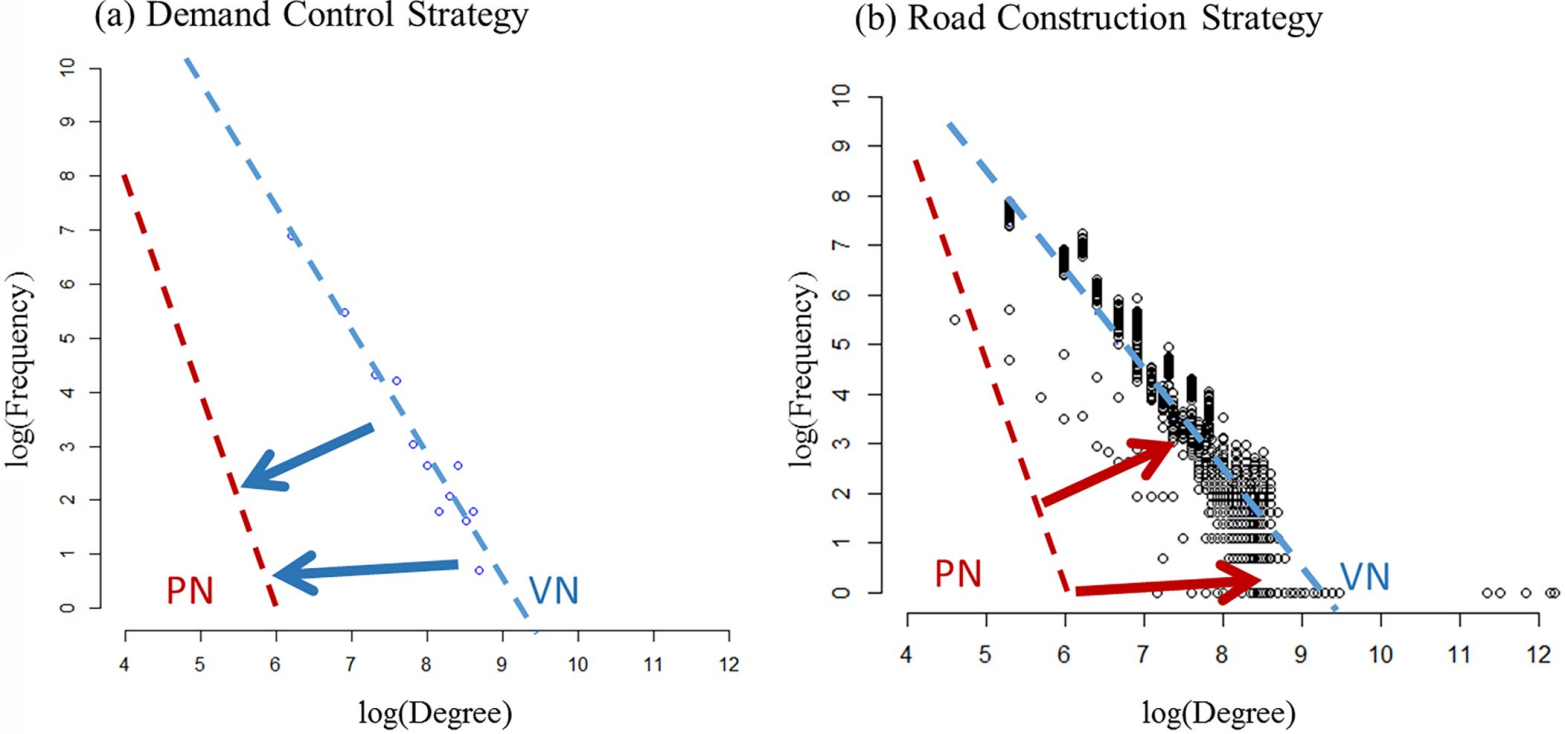
The application of proposed relation. (Drawn by authors using R program).

Fig 13 shows the possibility of applying the proposed evaluation measure to certain control methods for improving the network performance. When controlling the incoming vehicles in the TGs, we can lower the coefficient difference between the PN and VN by controlling the number of the incoming vehicles, as shown in Fig 13(A). The red dotted line represents the coefficient of the degree distribution of the PN, and the blue dotted line represents the coefficient of the degree distribution of the VN. We can improve the entire network’s performance not only by considering the local traffic status but also by considering the pattern of the OD demands of the entire network system. At the same time, the proposed measure can give a more efficient direction when planning to construct a new road. Fig 13(B) shows the aggregated degree distribution of the VN at all periods. There is a difference between the coefficient values of the two different networks as we can see in the figure. During the construction planning process, decisions can be made by checking if the coefficient difference would be lowered when the new road link is added to the existing network system. In this way, the performance of an entire road network can be improved efficiently.

## Conclusion

In this paper, we analyse the structural road network and hypothetical Origin-Destination demand network of Korea highway system from a point of view of the complex network theory. It is found that both networks follow the “power law” distribution in the weighted conditions. Particularly, in the case of the OD demand network, the coefficient of node degree distribution dynamically changes according to the time and it can also represent different travelling behaviours during weekdays and weekends.

By using the coefficient calculated from both weighted networks, we develop a network evaluation methodology. We analyse the effect of the total demand and coefficient difference of the structural network and OD demand network on the performance. The statistical analysis of the physical road network and demand network can be investigated with the difference between the designed properties of physical transportation network and desired properties of network structure perceived by road users. As we can see in the results, the total demand and coefficient difference show a positive linear relationship with respect to the network performance. Furthermore, a stronger linear relationship is observed when we consider the total demand and coefficient difference simultaneously. This means that not only the difference between the capacity of physical road network and the demand for road network but also the difference between the configuration of physical road network and distribution of origin-destination demand have significant influences on network efficiencies. Based on these results, we can effectively evaluate the performance of an entire highway network with a simple calculation method even in the environment in which the traffic demand continuously changes.

The focus of this paper is not only to analyse the relationship between physical road network and Origin-Destination demand network but also to provide a basis for further applications such as demand control for road network or planning of road network construction. It is shown that the proposed method is able to capture the network’s system-wide congestion caused by differences between characteristics of physical road networks and origin-destination demand networks. The proposed evaluation method can be used in two ways. First, it can give an efficient direction when modelling a control method to improve the performance of an entire road network. Second, it can also give an efficient direction when planning a new road construction. The proposed method that uses the relationship between the structural road network and OD network can provide the foundation for road controls and related policies in terms of improving the performance of road network at the state level.

Even if it is shown that the proposed method has a great advantage to evaluate the performance on the transportation network with a simple calculation and macroscopic perspective, the method is still required to be considered further for spatial information on the node distribution. Furthermore, since the ultimate goal of our proposed method is to provide a more efficient direction to the user, the effect of the proposed method on the urban structure and its future development may need to be further considered from the urbanization perspective. The recent researches such as [33,34] can be used for the network configuration setting by considering the spatial features in the urban area.
